# Achieving research competences through medical education

**DOI:** 10.1007/s40037-013-0084-x

**Published:** 2013-09-19

**Authors:** Friedo W. Dekker

**Affiliations:** Department of Clinical Epidemiology, Leiden University Medical Centre, Room C7-G 93, PO Box 9600, 2300 RC Leiden, the Netherlands

Recently, Van Schravendijk and colleagues published the results of a European survey on research components in medical curricula. The emerging picture shows wide variation between medical schools and between countries: 3/4 of the schools offer research courses and in 2/3 students can do research themselves. However, in most schools, less than 10 % of students choose this option. Only half the medical schools require the writing of any thesis or literature review for graduation [1]. In contrast, within the Netherlands there seems to be large agreement: all eight university medical centres spend a considerable amount of time (14–30 ECTS) on mandatory research courses, in addition to a full-time research project for at least 4 months that all medical students do before obtaining a Master’s degree [2].

Obviously, the Dutch 2009 Framework for Undergraduate Medical Education, specifying learning outcomes and competences and thereby almost serving as a blueprint for medical curricula in the Netherlands, has been indispensable in reaching this consensus [3]. However, there are many more considerations why this amount of time and effort is spent on academic and scientific training in medical curricula, as has been nicely put forward by De Beaufort and De Goeij [4] in this issue. Interestingly, as described by Marz et al. [5] also in Europe consensus in opinion about learning outcomes regarding scientific education seems to increase, although implementation in medical curricula across Europe is a challenging next step.

Some new approaches on implementing scientific training in medical education are presented in this issue. First, De Groot et al. [6] argue that it is useful to make a distinction between using evidence for individual patient care and using scientific knowledge for the development of protocols or guidelines for groups of patients or professionals. Making this distinction facilitates teaching of the accompanying competences and different CanMEDS roles. Secondly, Vereijken et al. [7] present an example where 300 second-year medical students all participated as researchers in a specifically designed collaborative study. They show that this active learning research experience—as part of just a 3-week course on epidemiology early in the curriculum—can foster student attitudes towards being more research-minded and, at the same time, can produce publishable results. Thirdly, Riley et al. [8] explain why and how they organized Self Selected Components where all students participate in at least four small-group collaborative research projects in their curriculum. Interestingly, they defined research more broadly, including literature reviews and clinical audit projects. Fourthly, several papers in this issue make a distinction between two separate aims in scientific education: to enable future doctors to ‘use’ research (in clinical practice) and to enable them (or at least stimulate them) to ‘do’ research (in academia or in other professional context). From this perspective, using research is related to evidence-based practice, and teaching involves critical appraisal and other classical EBM skills. Doing research, however, also involves other skills such as designing a study, performing statistical analyses, and writing a research paper. This distinction is also important in order to understand discussions about the design of a medical curriculum: is it sufficient for students to do a small literature study, or are they required to do a full empirical research project?

As far as the aim of teaching research skills is related to changing future doctors’ behaviour in using research and applying evidence-based practice, there is still a lot to gain. In her PhD thesis, Esther de Groot [9] argues that veterinarians might benefit from continued learning through critically reflective work behaviour in communities in order to support the transition to evidence-based practice. Zwolsman et al. [10] analysed 147 consultations by 34 general practitioners and concluded that EBM behaviour is difficult to observe in a GP’s daily practice. Both studies show that actually using research and communicating about it in clinical practice is not always easy, and a lot more research is necessary on this topic.

As far as the aim of teaching research skills is related to stimulating future doctors to do research, write a PhD thesis, and develop an academic career, it is fair to say that we do not have much evidence of the effectiveness of our educational efforts. But might that be an achievable goal for the next few years?

By and large, in this special issue of PME, scientific education is viewed from different angles, ranging from educational researchers to programme directors, from undergraduate education to specialist training, and from aiming at using research to actually doing research.
